# 955. Trends in Top COVID-19 Questions Among a National Audience of Primary Care Clinicians

**DOI:** 10.1093/ofid/ofab466.1150

**Published:** 2021-12-04

**Authors:** Aylin Madore, Margaret Oliverio, Steven Nock

**Affiliations:** DBC Pri-Med LLC, Boston, MA

## Abstract

**Background:**

As COVID-19 took the world by storm, primary care clinicians (PCCs) played a critical role in identification and management of this disease. Yet, knowledge around COVID-19 is constantly evolving, leaving clinicians with many unanswered questions. We sought to examine what questions PCCs had about COVID-19 and whether there were any trends over time.

**Methods:**

We sought to examine what questions PCCs had about COVID-19 and whether there were any trends over time. We collected questions from PCCs during 4 live virtual 60-minute continuing medical education (CME) panel discussions on COVID-19 led by infectious disease experts from November 2020 to February 2021. Questions were independently sorted and analyzed by 2 MDs using constant-comparison and tie-break methodology.

**Results:**

A total of 600 questions pertaining to COVID-19 were collected across 4 sessions. Top questions asked by PCCs ranked in descending order related to the following topics, with most common themes listed in parentheses: 1. Vaccines (efficacy, safety in pregnancy, indications/contraindications, timing of administration, side effects/adverse events) 2. Medication-Specific Treatment (monoclonal antibodies, ivermectin, steroids, convalescent plasma, supplements [vitamin D, zinc, vitamin c]) 3. Testing (false positive/false negatives, use in travel, quarantine, and gatherings) 4. Other Management (role of anticoagulation, use of chronic medications, guidelines) 5. Personal Protective Equipment (masks, eye protection, post-vaccination, use in travel). [Table 1] The percentage of questions around vaccination increased from 5% of total questions in October 2020 to 67% in February 2021. Questions related to Treatment declined from 20% to 6%, Testing declined from 21% to 3%, Other Management declined from 6% to 1% and PPE increased from 3% to 8% during this period.

Table 1. Top 5 topics of questions listed in descending order of frequency across all 4 COVID-19 panel sessions.

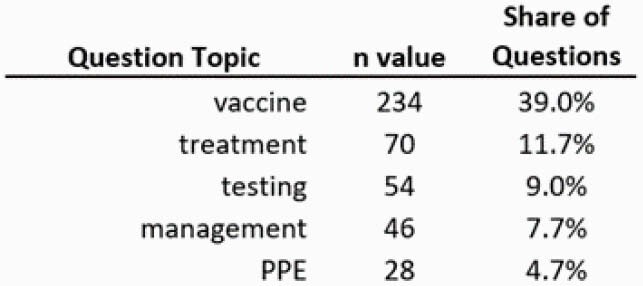

Table 2. Percentage of questions in the top 5 topics for each of the 4 COVID-19 panel sessions, with associated trendline.

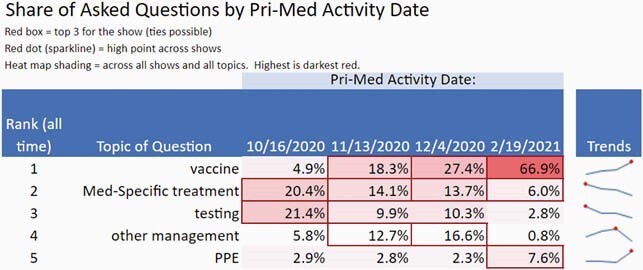

**Conclusion:**

PCCs nationally have gaps in knowledge around COVID-19 which can impact clinical decision-making. Based on our analysis of questions submitted by PCCs to infectious disease experts in a CME setting, the greatest gaps in knowledge were around vaccination, treatment, and testing with vaccination showing the greatest shift in interest over time.

**Disclosures:**

**All Authors**: No reported disclosures

